# Association of virtual end-of-life care with healthcare outcomes before and during the COVID-19 pandemic: A population-based study

**DOI:** 10.1371/journal.pdig.0000463

**Published:** 2024-03-13

**Authors:** John M. Lapp, Thérèse A. Stukel, Hannah Chung, Chaim M. Bell, R. Sacha Bhatia, Allan S. Detsky, James Downar, Sarina R. Isenberg, Douglas S. Lee, Nathan Stall, Peter Tanuseputro, Kieran L. Quinn

**Affiliations:** 1 Department of Medicine, Sinai Health System, Toronto, Canada; 2 Northern Ontario School of Medicine University, Sudbury, Canada; 3 ICES, Toronto and Ottawa, Canada; 4 Insitute of Health Policy, Management and Evaluation, University of Toronto, Toronto, Canada; 5 Department of Medicine, University of Toronto, Toronto, Canada; 6 Peter Munk Cardiac Centre and the Ted Rogers Centre for Heart Research, University Health Network, Toronto, Canada; 7 Division of Palliative Care, Department of Medicine, University of Ottawa, Ottawa, Canada; 8 Bruyere Research Institute, Ottawa, Canada; 9 Queensland University of Technology School of Law, Queensland, Australia; 10 Department of Family and Community Medicine, University of Toronto, Toronto, Canada; 11 Clinical Epidemiology Program, Ottawa Hospital Research Institute, Ottawa, Canada; 12 Temmy Latner Centre for Palliative Care, Sinai Health System, Toronto, Canada; Iran University of Medical Sciences, IRAN (ISLAMIC REPUBLIC OF)

## Abstract

The use of virtual care for people at the end-of-life significantly increased during the COVID-19 pandemic, but its association with acute healthcare use and location of death is unknown. The objective of this study was to measure the association between the use of virtual end-of-life care with acute healthcare use and an out-of-hospital death before vs. after the introduction of specialized fee codes that enabled broader delivery of virtual care during the COVID-19 pandemic. This was a population-based cohort study of 323,995 adults in their last 90 days of life between January 25, 2018 and December 31, 2021 using health administrative data in Ontario, Canada. Primary outcomes were acute healthcare use (emergency department, hospitalization) and location of death (in or out-of-hospital). Prior to March 14, 2020, 13,974 (8%) people received at least 1 virtual end-of-life care visit, which was associated with a 16% higher rate of emergency department use (adjusted Rate Ratio [aRR] 1.16, 95%CI 1.12 to 1.20), a 17% higher rate of hospitalization (aRR 1.17, 95%CI 1.15 to 1.20), and a 34% higher risk of an out-of-hospital death (aRR 1.34, 95%CI 1.31 to 1.37) compared to people who did not receive virtual end-of-life care. After March 14, 2020, 104,165 (71%) people received at least 1 virtual end-of-life care visit, which was associated with a 58% higher rate of an emergency department visit (aRR 1.58, 95%CI 1.54 to 1.62), a 45% higher rate of hospitalization (aRR 1.45, 95%CI 1.42 to 1.47), and a 65% higher risk of an out-of-hospital death (aRR 1.65, 95%CI 1.61 to 1.69) compared to people who did not receive virtual end-of-life care. The use of virtual end-of-life care was associated with higher acute healthcare use in the last 90 days of life and a higher likelihood of dying out-of-hospital, and these rates increased during the pandemic.

## Author summary

On March 14, 2020, the Government of Ontario introduced new specialized fee codes to support and incentivize the delivery of virtual care. Virtual care use increased substantially during the COVID-19 pandemic when people transitioned to staying home and remaining socially distant from one another. Evidence suggests the use of virtual care by people attending a walk-in clinic was associated with higher use of the emergency department. Healthcare at the end-of-life often includes frequent emergency department use and hospitalization. Given the high number of resources used for end-of-life care, we sought to study the relationship between virtual end-of-life care and acute healthcare use before and after the increase in virtual care provision due to COVID-19. We found that the use of virtual end-of-life care was associated with increased levels of emergency department use and hospitalizations, as well as a lower likelihood of dying in-hospital. These associations existed before and during the COVID-19 pandemic, but the magnitudes increased during the pandemic. As we try to understand the best uses for virtual care beyond the COVID-19 pandemic, our study provides insight into the acute healthcare use patterns and location of death for people who used virtual end-of-life care.

## Introduction

The COVID-19 pandemic necessitated restrictions on the delivery of in-person care to reduce the risk of SARS-CoV-2 transmission. These included direct instructions on March 15, 2020 from Ontario’s Chief Medical Officer of Health to decrease elective surgeries and nonemergent health services and then on March 19, 2020 to subsequently stop or reduce all nonessential or elective services, followed by a phased resumption of certain services and surgery [1,2]. More broadly, general messaging to the public was to stay home as much as possible and only attend essential services when needed, taking care to wear proper masks and socially distance whenever possible [2]. In an effort to adapt healthcare delivery in response to the pandemic, the provincial Government of Ontario introduced additional specialized fee codes on March 14, 2020, that enabled and incentivized broader delivery of virtual care across the healthcare system. Virtual care can be delivered via telephone, videoconference, secure email, and remote monitoring [3], and traditionally represented a small proportion of all care prior to the COVID-19 pandemic [1]. During the pandemic, virtual care and virtual end-of-life care use in Ontario rapidly increased [4,5]. Emerging evidence suggests that the use of virtual care for people attending a walk-in clinic during the pandemic was associated with higher use of the emergency department, which may depend upon the type of care received and the provider delivering it [6,7].

Health systems are increasingly focused on the delivery of high-value care that achieves desired health outcomes for the lowest possible cost [8]. When asked, many people indicate a preference to receive end-of-life care at home; a place most people also prefer to die [9,10]. These preferences are recognized such that an out-of-hospital death is used as a quality indicator for end-of-life care in many jurisdictions [10–13]. However, end-of-life care is associated with high healthcare costs, which is often related to frequent emergency department visits and hospitalizations [14,15]. What is not known is the associated impact of virtual care on these important end-of-life outcomes. As virtual end-of-life care becomes more accessible and widely used, it is essential to study its value as part of care delivery to the people who use it and the payers who reimburse it to inform ongoing coverage policies at a system level [16].

Given these knowledge gaps, the objective of this study was to measure the association between the use of virtual end-of-life care and acute healthcare use (emergency department, hospitalization) and an out-of-hospital death before and after the introduction of specialized fee codes that enabled broader delivery of virtual care during the COVID-19 pandemic. We hypothesized that virtual end-of-life care would be associated with lower rates of acute healthcare use and an increased likelihood of an out-of-hospital death, given that people receiving end-of-life care would have increased access to their physician via virtual care, wanted to avoid unnecessary exposure to SARS-CoV-2 during the pandemic, and generally prefer to die at home [10,17–19]. This study asks a novel research question that helps describe the impact of the COVID-19 pandemic on people at the end-of-life and may prompt further discussion on use-cases for virtual care.

## Methods

This study is reported in accordance with guidelines for The Reporting of studies Conducted using Observational Routinely-collected health Data (RECORD)[20].

### Study design, setting, and data sources

We conducted a population-based cohort study in Ontario, Canada using linked clinical and health administrative databases. These datasets were linked using unique encoded identifiers and analyzed at ICES (formerly the Institute for Clinical Evaluative Sciences). These datasets are commonly used to study end-of-life care [11,15](S2 Table).

Ontario has over 10 million adults, making it Canada’s largest province that accounts for nearly 40% of its national population. Ontario residents have access to medically necessary services via the Ontario Health Insurance Plan (OHIP), and those aged ≥65 years receive prescription drug insurance coverage via the Ontario Drug Benefit program [21].

ICES is an independent, non-profit research institute whose legal status under Ontario’s health information privacy law allows it to collect and analyze health care and demographic data, without consent, for health system evaluation and improvement. The use of the data in this project is authorized under section 45 of Ontario’s Personal Health Information Protection Act (PHIPA) and does not require review by a Research Ethics Board.

### Study cohort

Our study cohort included all Ontario adults (aged ≥18 years) in their last 90 days of life who died between January 25, 2018, and December 31, 2021. People were excluded from the study if on the 90^th^ day prior to death they were missing data on age or sex; aged <18 or >105 years; non-residents of Ontario; not eligible for OHIP for a period of ≥3 months in the previous year; did not have at least one healthcare encounter in the prior 10 years; resided in a nursing home; or were hospitalized during the entire period of follow-up between index and death date as they would not be eligible to receive virtual end-of-life care. Decedent cohorts are frequently used to study end-of-life care practices; by setting a standard lookback period in relation to death they do not rely on physician prognostication of survival [15,22,23].

### Use of Virtual End-of-Life Care (Exposure)

We measured exposure to virtual end-of-life care during the 90 days prior to death using a distinct set of specialized virtual care physician fee codes (S1 Table). People who received at least 1 virtual end-of-life care visit were assigned to the exposed group and their first virtual end-of-life care visit was the index study date. People who did not receive any virtual care visits in the last 90 days of life were assigned to the unexposed group and were randomly assigned an index study date based on the distribution of index dates from the exposed group. Prior to March 14, 2020, healthcare providers were reimbursed by OHIP for delivering virtual end-of-life care via a single telephone palliative care fee code. Providers could also be reimbursed through Ontario Telemedicine Network’s (OTN) distinct set of video-based fee codes, which required both the physician and patient to attend an authorized virtual care centre in person (S1 Table). On March 14, 2020, the Ontario Government introduced an additional set of specialized telephone and video-based provider fee codes to enable broader delivery of virtual care during the COVID-19 pandemic due to the restrictions on in-person clinical care. These codes removed the previous requirement for both parties to attend the appointment from an authorized virtual care centre and permitted the freedom to attend these appointments in any location using either the telephone or by video (S1 Table).

People were assigned to the “pre-March 14” or “post-March 14” intervention group based on their index date and date of death in relation to March 14, 2020 that allowed a focus on the environment in which someone received the majority of their care during the last 90 days of life. A person whose index and death dates both fell before March 14, 2020 was assigned to the pre-March 14 group, while a person whose index and death dates both fell after March 14, 2020 was assigned to the post-March 14 group. People whose index date came before, and death date came after March 14, 2020 were assigned to the pre-March 14 intervention group if their death occurred <45 days after March 14, 2020, and the post-March 14 intervention group if their death occurred ≥45 days after March 14, 2020.

### Patient characteristics

We measured multiple validated demographic and clinical variables at the index date including age, sex, socioeconomic status, surname-based ethnicity [24], rurality of residence, multiple chronic conditions [25–34], and Local Health Integrated Network (LHIN, now called the Home and Community Care Support Services [35]) organization based on residency. There are 14 LHINs/Home and Community Care Support Services organizations in Ontario that are responsible to plan and distribute provincial funding for regional public healthcare services. We used a 5-year look back period from index date to measure prevalent comorbidities and the hospital frailty risk score [36]. We recorded each person’s year and location of death, use of acute healthcare services and engagement with palliative care (using distinct provider fee codes[11,15,37,38]) in the one year prior to study index date, as well as the receipt of homecare in the two years prior to study index date. Homecare services are allocated based on needs identified by a homecare case manager, and may include longitudinal care with visits from allied healthcare providers including personal support workers, nurses, physiotherapists, occupational and speech-language therapists. These patient characteristics were measured because they were potential confounders between the association of virtual end-of-life care receipt and other acute health service use and were available in the ICES datasets.

### Outcomes

The primary outcome was emergency department visits or hospitalization for any cause in the last 90 days of life and after the index date. The secondary outcome was the location of death, categorized as occurring in- or out-of-hospital. Location of death was determined based on the database in which the death was registered (S2 Table).

### Statistical analysis

Multivariable modified Poisson regression measured the association of use of virtual end-of-life care with number of emergency department visits and hospitalizations in the period after the index date to death, where the logarithm of the follow-up period was used as an offset. It was also used to measure the association between virtual end-of-life care and out-of-hospital death. Use of modified Poisson regression allows estimation of relative rate which is more interpretable than odds ratios as well as including a robust standard error to account for overdispersion [39,40]. All models were adjusted for deciles of age, sex, neighbourhood income quintile, rural residence, LHIN, prevalent comorbidities, hospital frailty score, acute healthcare system use in the year prior to index date, and the receipt of homecare in the two years prior to index date. All analyses were performed using SAS version 9.4 (SAS Institute, Cary, North Carolina).

### Patient and public involvement

Patient and caregiver partners assisted in the development of the research questions and interpretation of the main findings.

## Results

### Characteristics of the study cohort

**Fig 1:**
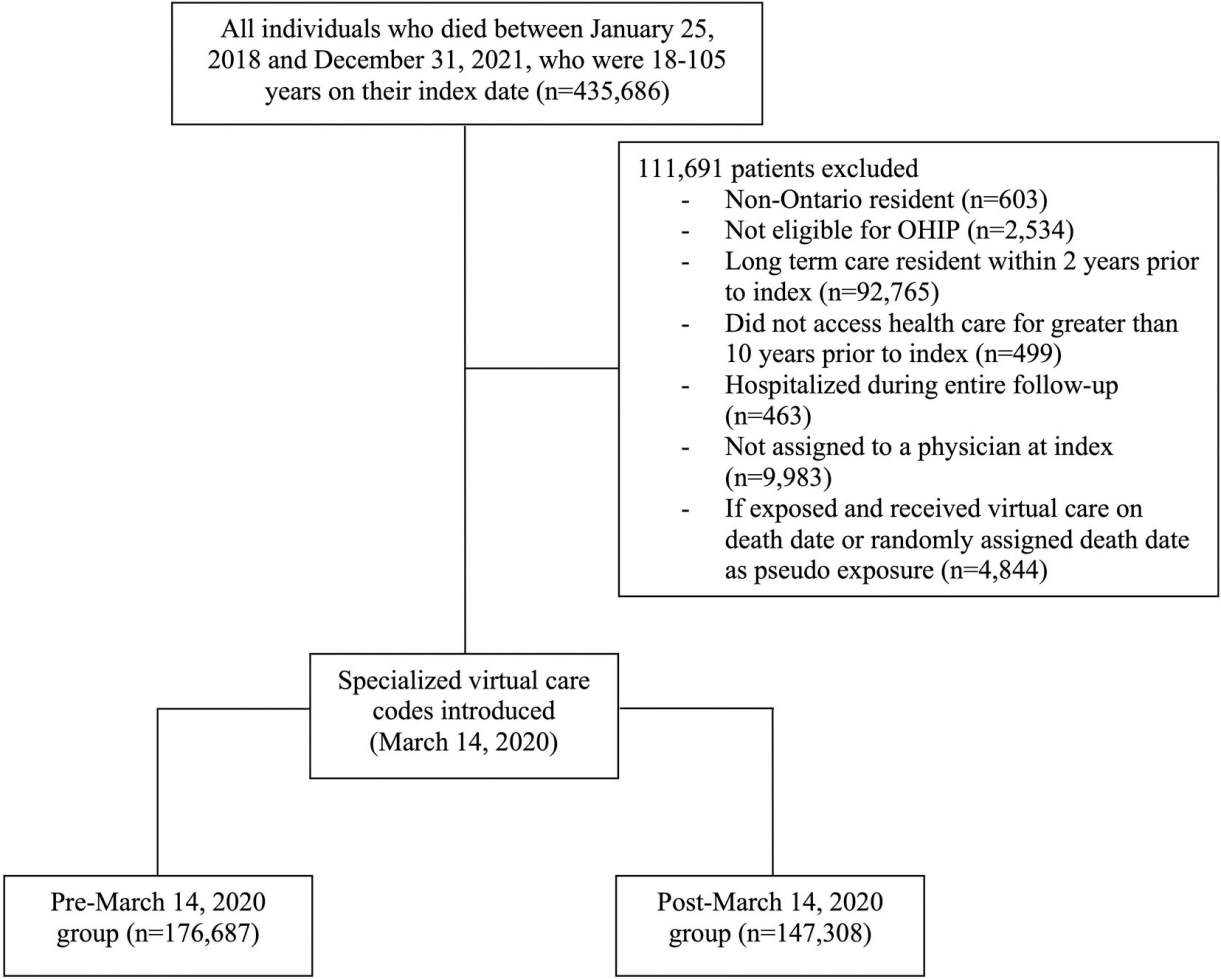
Creation of the study cohort.

The final study cohort included 323,995 adults; 176,687 people died before March 14, 2020, and 147,308 people died after (Fig 1). Prior to March 14, 2020, 13,974 people (7.9%) received virtual end-of-life care and 104,165 people (71%) received virtual end-of-life care after. The characteristics of people in the pre- and post-March 14 period are presented in Tables 1 and 2.

**Table 1:** Baseline characteristics of patients during their last 90 days of life, pre-March 14, 2020.

Variable	Exposed—received virtual end-of-life care	Unexposed—did not receive virtual end-of-life care	Standardized difference
	N = 13,974	N = 162,713	
Age, Mean ± SD	71.43 ± 15.39	74.92 ± 15.39	0.23
Female Sex, n (%)	6,324 (45.3%)	73,135 (44.9%)	0.01
Neighbourhood income quintile, n (%)			
1	3,389 (24.3%)	41,305 (25.4%)	0.03
2	2,997 (21.4%)	36,022 (22.1%)	0.02
3	2,723 (19.5%)	31,074 (19.1%)	0.01
4	2,409 (17.2%)	27,498 (16.9%)	0.01
5	2,410 (17.2%)	26,153 (16.1%)	0.03
Missing			
LHIN, n (%)			
Central	983 (7.0%)	17,414 (10.7%)	0.13
Central East	1,275 (9.1%)	19,471 (12.0%)	0.09
Central West	513 (3.7%)	7,631 (4.7%)	0.05
Champlain	1,327 (9.5%)	15,428 (9.5%)	0
Erie St. Clair	638 (4.6%)	9,572 (5.9%)	0.06
Hamilton Niagara Haldimand Brant	1,461 (10.5%)	21,453 (13.2%)	0.08
Mississauga Halton	678 (4.9%)	10,793 (6.6%)	0.08
North East	2,355 (16.9%)	7,777 (4.8%)	0.40
North Simcoe Muskoka	819 (5.9%)	6,797 (4.2%)	0.08
North West	772 (5.5%)	3,294 (2.0%)	0.18
South East	830 (5.9%)	7,865 (4.8%)	0.05
South West	1,048 (7.5%)	13,341 (8.2%)	0.03
Toronto Central	685 (4.9%)	13,248 (8.1%)	0.13
Waterloo Wellington	590 (4.2%)	8,629 (5.3%)	0.05
Surname-based ethnicity, n (%)			
Chinese	200 (1.4%)	4,102 (2.5%)	0.08
South Asian	201 (1.4%)	3,290 (2.0%)	0.04
General Population	13,573 (97.1%)	155,321 (95.5%)	0.09
Rural residence, n (%)	3,416 (24.4%)	19,627 (12.1%)	0.32
Missing	42 (0.3%)	594 (0.4%)	
Heart failure, n (%)	3,479 (24.9%)	49,618 (30.5%)	0.13
COPD, n (%)	3,260 (23.3%)	36,466 (22.4%)	0.02
Diabetes, n (%)	4,946 (35.4%)	61,387 (37.7%)	0.05
Cancer, n (%)	10,604 (75.9%)	106,711 (65.6%)	0.23
Dementia, n (%)	1,427 (10.2%)	25,072 (15.4%)	0.16
End-stage renal disease, n (%)	3,421 (24.5%)	46,021 (28.3%)	0.09
Severe liver disease, n (%)	395 (2.8%)	3,537 (2.2%)	0.04
Stroke, n (%)	1,374 (9.8%)	20,046 (12.3%)	0.08
Hypertension, n (%)	9,576 (68.5%)	121,027 (74.4%)	0.13
Psychotic disorder mental health (e.g., schizophrenia), n (%)	221 (1.6%)	3,166 (1.9%)	0.03
Non-psychotic mental health disorder (e.g., depression, anxiety), n (%)	3,746 (26.8%)	38,907 (23.9%)	0.07
Alcohol and substance use disorder, n (%)	1,348 (9.6%)	8,675 (5.3%)	0.16
Hospital frailty risk score, n (%)			
0. 0	1,995 (14.3%)	16,647 (10.2%)	0.12
1. 0.1–4.9	3,854 (27.6%)	37,486 (23.0%)	0.1
2. 5.0–8.9	1,939 (13.9%)	21,352 (13.1%)	0.02
3. 9.0 +	2,933 (21.0%)	38,994 (24.0%)	0.07
4. No hospitalizations	3,253 (23.3%)	48,234 (29.6%)	0.14
Number of emergency department visits in the year prior to index, Mean ± SD	2.04 ± 3.70	1.34 ± 2.78	0.21
Number of hospital admissions in the year prior to index, Mean ± SD	1.11 ± 1.45	0.91 ± 1.36	0.15
Received palliative care in the year prior to index, n (%)	2,797 (20.0%)	18,781 (11.5%)	0.23
Received long-term homecare in the 2 years prior to index, n (%)	6,561 (47.0%)	73,461 (45.1%)	0.04
Received designated end-of-life homecare in the 2 years prior to index, n (%)	4,216 (30.2%)	26,545 (16.3%)	0.33

**Table 2:** Baseline characteristics of patients during their last 90 days of life, post-March 14, 2020.

Variable	Exposed—received virtual end-of-life care	Unexposed—did not receive virtual end-of-life care	Standardized difference
	N = 104,165	N = 43,143	
Age, Mean ± SD	75.44 ± 14.35	72.67 ± 18.60	0.17
Female Sex, n (%)	47,785 (45.9%)	18,095 (41.9%)	0.08
Neighbourhood income quintile, n (%)			
1	24,668 (23.7%)	12,814 (29.7%)	0.14
2	22,893 (22.0%)	9,577 (22.2%)	0.01
3	20,551 (19.7%)	7,969 (18.5%)	0.03
4	18,151 (17.4%)	6,516 (15.1%)	0.06
5	17,523 (16.8%)	5,944 (13.8%)	0.08
Missing	379 (0.4%)	323 (0.7%)	0.05
LHIN, n (%)			
Central	12,489 (12.0%)	3,541 (8.2%)	0.13
Central East	12,777 (12.3%)	4,730 (11.0%)	0.04
Central West	6,085 (5.8%)	1,556 (3.6%)	0.11
Champlain	9,495 (9.1%)	4,039 (9.4%)	0.01
Erie St. Clair	5,692 (5.5%)	2,786 (6.5%)	0.04
Hamilton Niagara Haldimand Brant	12,943 (12.4%)	5,605 (13.0%)	0.02
Mississauga Halton	7,805 (7.5%)	2,066 (4.8%)	0.11
North East	4,958 (4.8%)	3,223 (7.5%)	0.11
North Simcoe Muskoka	4,407 (4.2%)	1,931 (4.5%)	0.01
North West	1,757 (1.7%)	1,479 (3.4%)	0.11
South East	4,476 (4.3%)	2,390 (5.5%)	0.06
South West	7,738 (7.4%)	4,029 (9.3%)	0.07
Toronto Central	8,076 (7.8%)	3,555 (8.2%)	0.02
Waterloo Wellington	5,467 (5.2%)	2,213 (5.1%)	0.01
Surname-based ethnicity, n (%)			
Chinese	3,129 (3.0%)	944 (2.2%)	0.05
South Asian	2,741 (2.6%)	683 (1.6%)	0.07
General Population	98,271 (94.3%)	41,510 (96.2%)	0.09
Missing	24 (0.0%)	6 (0.0%)	0.01
Rural residence, n (%)	11,791 (11.3%)	6,543 (15.2%)	0.11
Missing	352 (0.3%)	292 (0.7%)	0.05
Heart failure, n (%)	30,009 (28.8%)	10,175 (23.6%)	0.12
COPD, n (%)	21,591 (20.7%)	7,397 (17.1%)	0.09
Diabetes, n (%)	41,700 (40.0%)	14,308 (33.2%)	0.14
Cancer, n (%)	72,742 (69.8%)	21,222 (49.2%)	0.43
Dementia, n (%)	15,549 (14.9%)	7,806 (18.1%)	0.09
End-stage renal disease, n (%)	29,514 (28.3%)	9,641 (22.3%)	0.14
Severe liver disease, n (%)	1,920 (1.8%)	417 (1.0%)	0.07
Stroke, n (%)	11,837 (11.4%)	4,931 (11.4%)	0
Hypertension, n (%)	78,702 (75.6%)	28,907 (67.0%)	0.19
Psychotic disorder mental health (e.g., schizophrenia), n (%)	1,889 (1.8%)	1,375 (3.2%)	0.09
Non-psychotic mental health disorder (e.g., depression, anxiety), n (%)	26,805 (25.7%)	9,725 (22.5%)	0.07
Alcohol and substance use disorder, n (%)	4,959 (4.8%)	4,499 (10.4%)	0.22
Hospital frailty risk score, n (%)			
0. 0	12,165 (11.7%)	3,201 (7.4%)	0.15
1. 0.1–4.9	24,264 (23.3%)	7,529 (17.5%)	0.15
2. 5.0–8.9	13,578 (13.0%)	4,642 (10.8%)	0.07
3. 9.0 +	23,038 (22.1%)	9,180 (21.3%)	0.02
4. No hospitalizations	31,120 (29.9%)	18,591 (43.1%)	0.28
Number of emergency department visits in the year prior to index, Mean ± SD	1.19 ± 2.35	0.98 ± 2.84	0.08
Number of hospital admissions in the year prior to index, Mean ± SD	0.82 ± 1.28	0.53 ± 1.15	0.23
Received palliative care in the year prior to index, n (%)	10,074 (9.7%)	2,066 (4.8%)	0.19
Received long-term homecare in the 2 years prior to index, n (%)	45,849 (44.0%)	15,466 (35.8%)	0.17
Received designated end-of-life homecare in the 2 years prior to index, n (%)	15,019 (14.4%)	2,892 (6.7%)	0.25

The characteristics of people receiving virtual end-of-life care changed in the pre- to post-March 14, 2020 period. Compared to people who did not receive virtual end-of-life care, people who received virtual end-of-life care and died prior to March 14, 2020 were generally younger, had more emergency department visits and hospitalizations in the last year. A higher proportion lived in a rural area, lived in the North East and North West LHINs, had cancer and alcohol/substance use disorders, and received palliative care in the prior year compared to people who did not receive virtual end-of-life care. A lower proportion of people who received virtual end-of-life care had heart failure, dementia, and hypertension, and lived in the Central and Toronto Central LHINs.

In contrast, after March 14, 2020, people who received virtual end-of-life care were generally older and had more hospitalizations in the last year than those who did not receive virtual end-of-life care. A lower proportion lived in a rural area, lived in the North East or North West LHINs, lived in a neighbourhood in the bottom income quintile, or had alcohol/substance use disorders, compared to people who did not receive virtual end-of-life care. A higher proportion of people who received virtual end-of-life care received palliative care in the prior year, homecare in the prior two years, and had heart failure, diabetes, cancer, end-stage renal disease, and hypertension, and lived in the Central, Central West, and Mississauga Halton LHINs (Tables 1 and 2).

### Virtual End-of Life care and acute healthcare use

**Fig 2:**
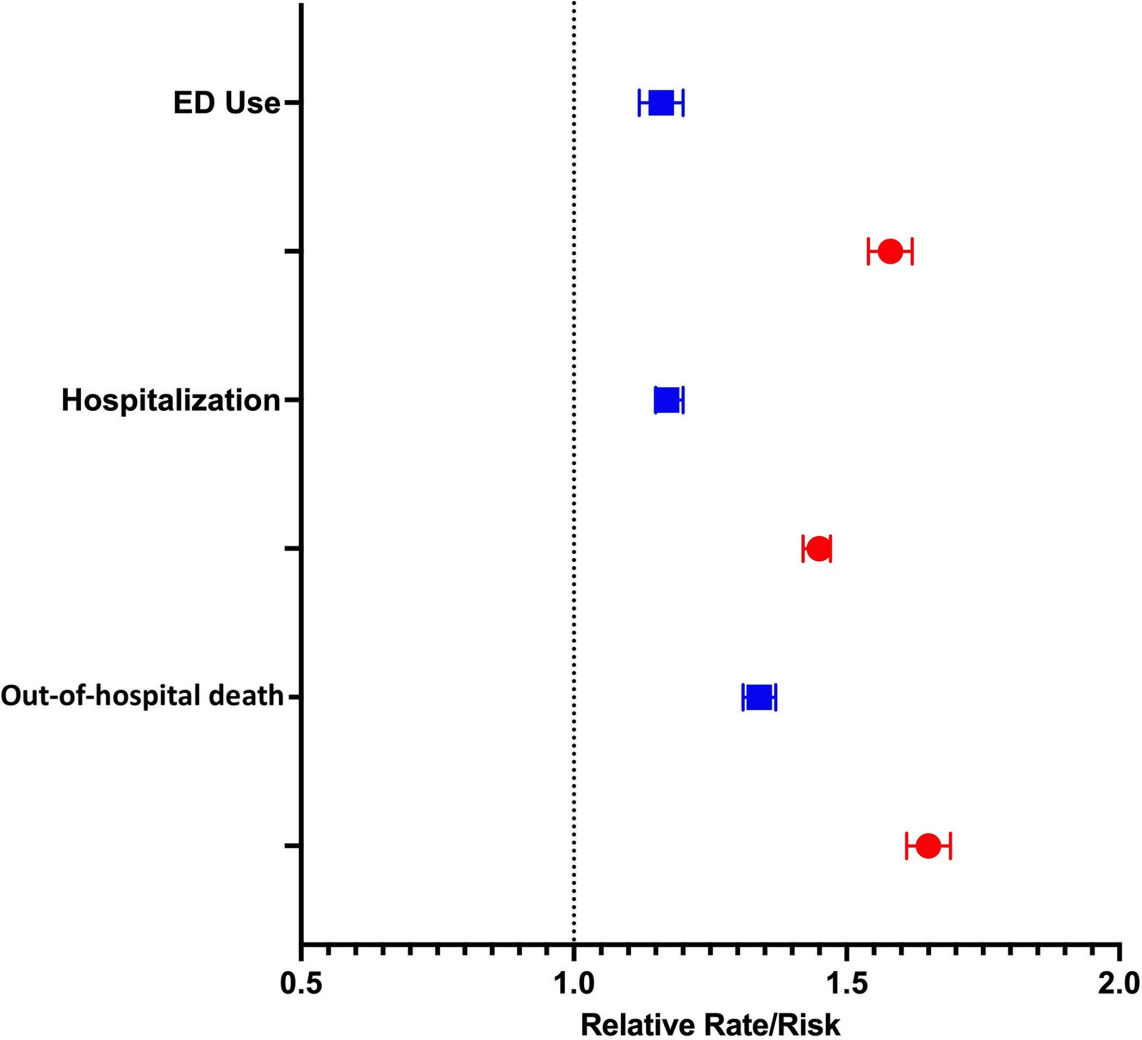
Forest plot of the adjusted relative risk of the association between the receipt of virtual end-of-life care and health service use (emergency department visits and hospitalizations) and out-of-hospital death for the pre-March 14, 2020 (blue squares) and post-March 14, 2020 (red circles) groups.

Prior to March 14, 2020, the use of virtual end-of-life care was associated with a 16% higher rate of emergency department use (Adjusted Relative Rate (aRR) 1.16, 95%CI 1.12 to 1.20) and a 17% higher rate of hospitalization (aRR 1.17, 95%CI 1.15 to 1.20) in the last 90 days of life, compared with people who did not receive virtual end-of-life care (Fig 2 and Table 3). After March 14, 2020, its use was associated with a 58% higher rate of emergency department use (aRR 1.58, 95%CI 1.54 to 1.62) and a 45% higher rate of hospitalization (aRR 1.45, 95%CI 1.42 to 1.47) in the last 90 days of life, compared with people who did not receive virtual end-of-life care (Fig 2 and Table 3).

**Table 3:** Association between the receipt of virtual end-of-life care and health service use (emergency department visits and hospitalizations) and out-of-hospital death §.

Outcome	Crude Rate	Adjusted Relative Rate (95% CI)
Pre-March 14, 2020		
Emergency Department	Per person 90 days Exposed: 1.03 Unexposed: 0.73	1.16 (1.12, 1.20) (Adjusted Relative Rate)
Hospitalization	Per person 90 days Exposed: 1.53 Unexposed: 1.36	1.17 (1.15, 1.20) (Adjusted Relative Rate)
Out-of-Hospital Death	N, (%) Exposed: 4,768, 34% Unexposed: 34,268, 21%	1.34 (1.31, 1.37) (Adjusted Relative Risk)
Post-March 14, 2020		
Emergency Department	Per person 90 days Exposed: 0.69 Unexposed: 0.48	1.58 (1.54, 1.62) (Adjusted Relative Rate)
Hospitalization	Per person 90 days Exposed: 1.25 Unexposed: 0.83	1.45 (1.42, 1.47) (Adjusted Relative Rate)
Out-of-Hospital Death	N, (%) Exposed: 31,854, 31% Unexposed: 6,459, 15%	1.65 (1.61, 1.69) (Adjusted Relative Risk)

§ All models were adjusted for deciles of age, sex, neighbourhood income quintile, rural residence, LHIN, prevalent comorbidities, hospital frailty risk score, acute healthcare system use in the year prior to index date, and the receipt of homecare in the two years prior to index date.

### Location of death

Receiving virtual end-of-life care before March 14, 2020 was associated with a 34% higher risk of an out-of-hospital death (aRR 1.34, 95%CI 1.31 to 1.37) (Fig 1 and Table 3). Receiving virtual end-of-life care after March 14, 2020 was associated with a 65% higher risk of an out-of-hospital death (aRR 1.65, 95%CI 1.61 to 1.69) (Fig 2 and Table 3).

## Discussion

This population-based cohort study of 323,995 adults who died in Ontario, Canada before or during the COVID-19 pandemic found that the use of virtual end-of-life care in the last 90 days of life was associated with an increase in emergency department use and hospitalizations, while simultaneously being associated with an increased likelihood of an out-of-hospital death. However, the magnitude of these associated effects appeared larger during the COVID-19 pandemic, which may be related to different characteristics of people in the pre- and post-March 14 groups as well as the introduction of restrictions on in-person care and the introduction of additional specialized fee codes to enable broader delivery of virtual care. While the contributory effects of the COVID-19 pandemic on patterns of healthcare use and delivery cannot be separated, the consistency of the associations across the pre- and post-March 14 periods strengthens our confidence in the findings. Strengths of this study include the use of linked clinical and health administrative databases, which represent experiences from across the province of Ontario as well as offer the ability to compare care before and after the introduction of additional specialized fee codes from the Government of Ontario.

A higher proportion of people who received virtual end-of-life care had cancer as well as prior healthcare use and involvement of palliative care compared to people who did not receive virtual end-of-life care. These people likely had a higher intensity of unmeasured healthcare needs such that virtual care was one method to identify them and ensure they accessed care in an appropriate setting. The different characteristics of people in the pre- and post-March 14 groups may explain the increased magnitude of effect that was observed given the post-March 14 group was generally older and sicker, which may further reflect residual confounding related to unmeasured care needs. People in the pre-March 14 group were younger and lived more rurally with high proportions of cancer and higher previous acute healthcare use and palliative care involvement. Virtual care in this timeframe was likely used to access people in remote regions with active cancer who may have been unsupported adequately at home by palliative care. Conversely, people in the post-March 14 group were older and lived in urban centres with high proportions of cancer and other significant comorbidities associated with high healthcare use. Under pandemic restrictions, virtual care likely identified those with unmet healthcare needs that may have gone undetected until much later when they ultimately presented to acute care settings. Residual confounding due to pandemic-related effects in healthcare use and delivery may have contributed to and/or modified the magnitude of the association.

The results from this population-based study builds on prior work reporting that virtual care achieved favourable outcomes for people receiving end-of-life care at home, especially as a supplement to in-person care [41–43]. The results also build on work published recently in Ontario that found the percentage of care a family physician provides virtually does not impact ED visit rates, while virtual walk-in visits increase the likelihood of an ED visit [6,7]. High value care that improves outcomes at the same or lower costs benefit both the patient and the health system [44]. It has been suggested that high value end-of-life care should deliver care at the right time and place for the person receiving it [45,46]. The intensity of care is known to significantly increase near the end-of-life, which frequently requires care in an emergency department or hospital setting [14]. Here, it is possible that persons using virtual end-of-life care did so because of unmet healthcare needs–the same needs that caused them to later utilize acute care. It may have also facilitated care at home at the very end-of-life through involvement of palliative care, frequent communication and monitoring clinical status to support people to die in a place most prefer [10,15,22]. Future research is needed to examine the complex interplay between individual patient preferences, needs, and the use of virtual end-of-life care. This work will serve as an essential component to inform continued use of virtual care, including virtual end-of-life care.

### Limitations

The use of administrative data limits our ability to measure patient preferences for location of care, modes of care delivery including virtual end-of-life care and location of death, which are essential components of high-quality end-of-life care. However, prior evidence reported that most people generally prefer to die at home [10]. Additionally, it is important to reiterate that these fee codes are not exclusively palliative in nature, and our definition of virtual end-of-life care is based on care provided in the last 90 days of life. The use of a decedent study design may introduce selection bias given that one may not know whether a potential recipient of virtual care would be alive after 90 days. However, decedent cohorts are frequently used to study end-of-life care practices and delivery [15,45]. They offer advantages such as not relying on physician prognostication of survival and instead setting a standard lookback period in relation to death [23]. Additionally, this study did not exclude specific causes of death and therefore should be representative of broad disease categories with varying levels of healthcare use required in the last 90 days of life. Death also serves as a common denominator that marks the seriousness of disease and avoids decision making about stopping follow up of a prospective cohort study [23]. Second, this data does not include information on the cause of death (including COVID-19) or the specific care needs of the person. The use of acute healthcare services in our study may reflect an underlying need for these care settings. Conversely, it may also reflect a lack of access to home-based care or differences in individual preferences for care setting. Third, virtual care is a broad term that includes delivery by telephone, videoconference, secure email, and remote monitoring, though none of the fee codes specifically indicate secure email or remote monitoring so it is unlikely those modalities were used in the context of this study (S1 Table) [3]. We were not able to distinguish whether specific types of virtual end-of-life care (e.g., telephone vs. video) were differentially associated with acute healthcare use, which may help inform which modality may be optimal for specific types of care delivery. Additionally while it may represent a small number of people overall, the magnitude of virtual end-of-life care use pre-March 14, 2020 may be overestimated if people did not attend their appointments as there are fee codes for missed appointments or those abandoned for technical difficulties (S1 Table). Fourth, we were not able to categorize whether people were accessing care through their family physician or through a walk-in clinic. It has been suggested that patients of family physicians who provided a high proportion of care virtually during the COVID-19 pandemic did not have higher levels of emergency department visits than those who provided the lowest levels of virtual care [7]. Conversely, people attending virtual walk-in clinics during the COVID-19 pandemic were more likely to visit the emergency department than those who saw their family physician virtually [6]. Fifth, the COVID-19 pandemic resulted in major shifts in healthcare delivery. Thus, the generalizability of our findings to future states is uncertain [47]. However, the observed consistency of our findings in the pre- and post-March 14 periods strengthens our confidence in these associations. By accounting for potential COVID-19 pandemic-related changes (comparing pre- and post-March 14), our results reflect changes during the COVID-19 pandemic and indicate associations, not causation. Finally, Ontario exists within a high-income nation that provides publicly funded healthcare. The generalizability of these findings to other regions without publicly funded healthcare, or to middle- and low-income nations where access to healthcare may be different is unknown.

## Conclusion

This study demonstrates that persons using virtual end-of-life care tend also to use more acute care and die at home compared with non-users of virtual care. The magnitude of this association increased following the introduction of specialized fee codes that enabled its broader use during the COVID-19 pandemic.

## Supporting information

S1 Table List of virtual care fee codes according to COVID-19 pandemic time periods. (DOCX)

S2 Table List and description of databases used. (DOCX)
