# Supplementary material for: Association of virtual end-of-life care with healthcare outcomes before and during the COVID-19 pandemic: A population-based study
Source: PLOS Digit Health. 2024 Mar 13;3(3):e0000463. doi: 10.1371/journal.pdig.0000463 (PMC10936771; doi:10.1371/journal.pdig.0000463)
Supplement: S1 Table — (DOCX) [file pdig.0000463.s001.docx]

**S1 Table: List of virtual care fee codes according to COVID-19 pandemic time periods.**

| **Pre-Pandemic Fee Codes (Pre-March 14, 2020)** | |
| --- | --- |
| B099A | Tracking Code |
| B100A | First Telemedicine Patient Encounter premium |
| B101A | First Cancelled/Missed Telemedicine Patient Encounter premium |
| B102A | First Technical Difficulties Abandoned Patient Encounter premium |
| B200A | Subsequent Telemedicine Patient Encounter premium |
| B201A | Subsequent Missed/Cancelled Telemedicine Patient Encounter premium |
| B202A | Subsequent Technical Difficulties Abandoned Patient Encounter premium |
| G511A | Telephone management regarding a patient receiving palliative care at home |

| **Pandemic Fee Codes (Post-March 14, 2020)** | |
| --- | --- |
| K080A | Minor assessment of a patient by telephone or video or advice or information by telephone or video to a patient’s representative regarding health maintenance, diagnosis, treatment and/or prognosis. |
| K081A | a. Intermediate assessment of a patient by telephone or video, or advice or information by telephone or video to a patient’s representative regarding health maintenance, diagnosis, treatment and/or prognosis, if the service lasts a minimum of 10 minutes; or  b. Psychotherapy, psychiatric or primary mental health care, counselling or interview conducted by telephone or video, if the service lasts a minimum of 10 minutes. |
| K082A | Psychotherapy, psychiatric or primary mental health care, counselling or  interview conducted by telephone or video per unit (unit means half hour or major part thereof). |
| K083A | Specialist Consultations and Visits by telephone or video. |
| B203A | Synchronous video visits with a patient in the home or another location of their choice (i.e. the patient is not at a patient host site). |
| G511A | Telephone management regarding a patient receiving palliative care at home |
